# Predicting treatment dropout after antidepressant initiation

**DOI:** 10.1038/s41398-020-0716-y

**Published:** 2020-02-06

**Authors:** Melanie F. Pradier, Thomas H. McCoy Jr, Michael Hughes, Roy H. Perlis, Finale Doshi-Velez

**Affiliations:** 1 Harvard John A. Paulson School of Engineering and Applied Sciences, 29 Oxford Street, Cambridge, MA 02138 USA; 2 Center for Quantitative Health, Massachusetts General Hospital, 185 Cambridge Street, Boston, MA 02114 USA; 3 Harvard Medical School, 25 Shattuck Street, Boston, MA 02115 USA; 4 Tufts University, 419 Boston Avenue, Medford, MA 02155 USA

**Keywords:** Psychology, Depression

## Abstract

Antidepressants exhibit similar efficacy, but varying tolerability, in randomized controlled trials. Predicting tolerability in real-world clinical populations may facilitate personalization of treatment and maximize adherence. This retrospective longitudinal cohort study aimed to determine the extent to which incorporating patient history from electronic health records improved prediction of unplanned treatment discontinuation at index antidepressant prescription. Clinical data were analyzed from individuals from health networks affiliated with two large academic medical centers between March 1, 2008 and December 31, 2014. In total, the study cohorts included 51,683 patients with at least one International Classification of Diseases diagnostic code for major depressive disorder or depressive disorder not otherwise specified who initiated antidepressant treatment. Among 70,121 total medication changes, 16,665 (23.77%) of them were followed by failure to return; maximum risk was observed with paroxetine (27.71% discontinuation), and minimum with venlafaxine (20.78% discontinuation); Mantel–Haenzel *χ*^2^ (8 df) = 126.44, *p* = 1.54e–23 <1e–6. Models incorporating diagnostic and procedure codes and medication prescriptions improved per-medication Areas Under the Curve (AUCs) to a mean of 0.69 [0.64–0.73] (ranging from 0.62 for paroxetine to 0.80 for escitalopram), with similar performance in the second, replication health system. Machine learning applied to coded electronic health records facilitates identification of individuals at high-risk for treatment dropout following change in antidepressant medication. Such methods may assist primary care physicians and psychiatrists in the clinic to personalize antidepressant treatment on the basis not solely of efficacy, but of tolerability.

## Introduction

While efforts at personalization of antidepressant (AD) treatment have focused on therapeutic efficacy, on average, modern ADs show greater differences in tolerability. For example, a recent large meta-analysis found significant differences for rates of acute discontinuation, but not efficacy, among 21 treatments investigated in randomized, controlled trials^1^. These authors highlighted the need to develop new strategies to distinguish individual-level differences in medication response, even where group-level differences are modest.

From a clinical perspective, personalization within medication class is challenging^2,3^. Treatment discontinuation may reflect a range of features, from depression-associated amotivation and hopelessness to failure to perceive a benefit to concerns about cost. However heterogeneous, the consequences of treatment discontinuation are substantial, contributing to poor treatment outcomes and depression chronicity^4^. Consistent with the importance of discontinuation as a clinical indicator, time to discontinuation has been used as an endpoint in large clinical trials^5^.

Previous work has demonstrated that electronic health records (EHRs) can be leveraged to generate sufficient sample sizes to facilitate machine learning studies of AD^6^. Here, we apply these methods to develop predictions of treatment discontinuation using large-scale EHR data from one health system and further characterize the performance of these models in a second academic medical center network. Specifically, we develop predictors of failure to return for a follow-up psychiatric visit of any kind after a change in antidepressant prescription as a face-valid, if nonspecific, adverse outcome readily detectable using coded EHR data.

## Methods

### Study overview and cohort description

The study cohort derivation began with 252,351 patients drawn from two academic medical center treatment networks in the Northeast United States (subsequently referred to as Site A and Site B) who received at least one antidepressant prescription between 2008 and 2014. The cohort included all individuals with at least one International Classification of Diseases, Ninth Revision (ICD9) diagnostic code for major depressive disorder (ICD9s 296.2x, 296.3x) or depressive disorder not otherwise specified (311). For these individuals, a datamart was generated using i2b2 server software (i2b2, Boston, MA, USA)^7^. Available patient data included sociodemographic information, diagnostic and procedure codes, as well as inpatient medication administrations and outpatient medication prescriptions extracted by ingredient and duration. The Partners HealthCare institutional review board approved the study protocol, waiving the requirement for informed consent as only de-identified data was utilized and no human subjects contact was required.

### Inclusion criteria

For model development, we restricted the cohorts to patients of age 18–80 years who were affiliated with one primary site (either Site A or B) and had received at least one of the nine most commonly-prescribed antidepressants (see Supplementary Table 1 for the list of antidepressants and Supplementary Fig. 1 for an illustration of this process). Individuals were excluded from primary analysis if they had no encounter of *any* type in the EHR system after 90 days from the last prescription registered (i.e., were potentially lost to follow-up, as could be the case if they transferred their care to another health system).

### Outcome definition

The primary outcome was treatment discontinuation following index prescription, defined as <90 days of prescription availability *and* no evidence of non-pharmacologic psychiatric treatment. This latter feature is defined as an absence of a psychiatric Current Procedural Terminology (CPT) code in 13 months following the last antidepressant prescription (see Supplementary Table 2 for a list of psychiatric codes), as a means of excluding individuals who, while not able to continue pharmacologic treatment, remain in ongoing psychiatric treatment. As noted above, we also excluded individuals who had *no* interaction with the health system in the 90 days following the last antidepressant treatment, as a means of decreasing the possibility that individuals were lost to follow-up because of (for example) change in treatment network or other transfer of care. Supplementary Fig. 2 illustrates the codes observed for an example individual discontinuing treatment after 225 days; of note, while there are no further antidepressant prescriptions or psychiatric visits, the continuation of non-psychiatric follow-up indicates that this individual has not been lost to follow-up.

For subsequent analysis, 27,366 eligible patients in the Site A cohort were randomly assigned to a training (80%), validation (10%), and testing (10%) data set. All 16,630 eligible patients from Site B were held out for testing.

### Modeling approach

#### Prediction task

We sought to predict treatment discontinuation for a given antidepressant medication on the basis of sociodemographic features, diagnostic codes, procedures, and medication data available *at the time of index prescription* of any given antidepressant, censoring all subsequent data. We therefore built models and evaluated the prediction task for each of the nine most-prescribed medications (see Supplementary Table 1) individually. Given a specific patient encounter and a specific medication, each prediction method yields a score or probability that the specific medication in question would lead to treatment discontinuation. We evaluated our prediction models at every change in treatment prescription under the condition that there was sufficient prior history—i.e., at least one prior ‘fact’ of any kind, comprised of diagnostic code, procedure, or prescription.

#### Feature derivation

From an initial set of 23,949 possible ICD or CPT codes and medications, we applied frequency thresholding to select 3852 codes occurring in at least 100 patients in Site A. For each patient and each encounter date, we built a compact representation of past event history via a count vector indicating how often each of these codewords appeared in the patient’s past history. Sociodemographic variables, including self-reported gender, race/ethnicity, and age at treatment event (in fractional years, e.g., 36.7 years old) are recorded for all patients at time of prescription and represented via one-hot encoded vectors (i.e., using binary features). We also include the calendar date of the treatment date (in fractional years) to capture secular trends.

#### Classification methods and metrics

We applied two standard classifiers, logistic regression (LR) and random forests (RF). Logistic regression used the implementation provided in the open-source Scikit Learn (version 0.18.1) toolkit for Python^8^. We tuned two hyperparameters on the Site A validation set: the type of regularization (L1 or L2 norm penalty) and the regularization strength. Random forests used the “ExtraTreeClassifier” implementation in Scikit Learn and tuned three hyperparameters on validation data: the number of trees, the fraction of features used in each tree, and the minimum number of samples at leaf nodes. Using each method, a separate classifier for each of the nine target medications was trained on the Site A training set. Hyperparameters were tuned using grid search to find the parameter combination that performs best on the Area Under the Curve (AUC) metric.

In primary analysis, model performance was compared using AUC for each of the nine medication prediction tasks in the held-out testing set from Site A and then in the independent Site B, to understand the extent to which code-based prediction of treatment discontinuation generalize to different scenarios. Recognizing that there are numerous means of model comparison, we elected to compare AUC’s using Student’s *t*-tests for familiarity and simplicity^9^. In light of the large cohort as well as the need to test multiple hypotheses simultaneously, we conservatively chose to apply Bonferroni correction by considering a significance level alpha = 0.05/100 = 0.0005 for these comparisons. Statistical validation for AUC values is reported in Supplementary Table 3 using 100 bootstraps. In secondary analysis, we characterized the performance of the two classifiers in sub-cohorts. First, while primary analysis sought to model all providers jointly, we subsequently examined models restricted to psychiatric versus non-psychiatric prescribers in order to address the possibility that each setting might have more prescriber-specific predictors. Second, we examined performance of models stratified by number of prior failed treatments—i.e., to what extent does ability to predict dropout *change* with additional treatment data.

## Results

### Rates of dropout across provider types and demographic subgroups

**Fig. 1:**
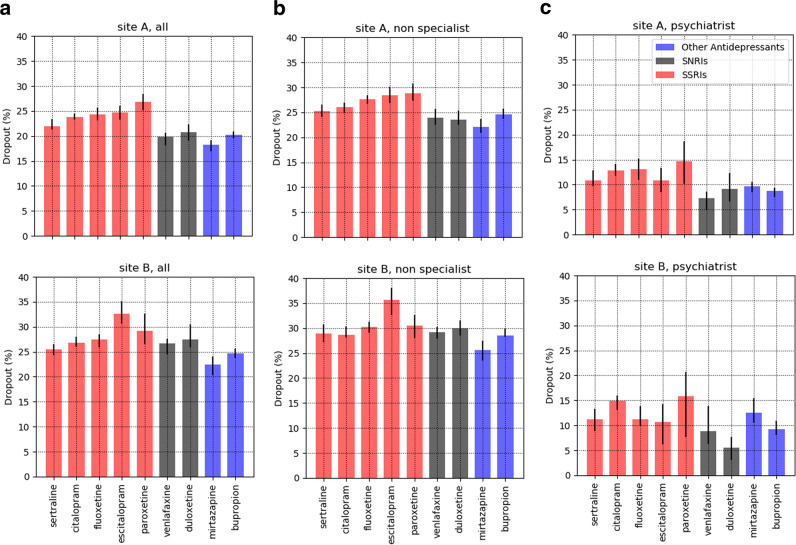
Dropout rates among all index prescriptions between 2008 and 2014. From left to right: **a** all prescriptions; **b** prescriptions provided by non-specialists; **c** prescriptions provided by psychiatrists. SNRIs: serotonin and norepinephrine reuptake inhibitors, SSRIs: selective serotonin reuptake inhibitors.

The two cohorts, spanning Site A and Site B, included 43,996 patients of which 14,975 (34.04%) were male, 34,544 (78.52%) were white, and 27,366 (62.20%) were cared for at Site A; mean age was 47.7 (SD 15.4) years. Demographics for the cohort by site are shown in Table 1. In total, 9502 of 27,366 (34.7%) patients from Site A discontinued treatment after a new antidepressant prescription, and 6274 of 16,630 (37.7%) at Site B. Figure 1 shows the proportion of dropouts among each antidepressant, organized by pharmacologic class. Among 70,121 total medication changes, 16,665 (23.77%) of them were followed by failure to return; maximum risk was observed with paroxetine (27.71% discontinuation), and minimum with venlafaxine (20.78% discontinuation). In general, greatest rates of discontinuation were observed with selective serotonin reuptake inhibitors across all types of providers (panel a), and discontinuation rates among non-psychiatrists (panel b) were substantially greater than psychiatrists (panel c); median Mantel–Haenzel *χ*^2^ (1 df) = 150.58, *p* = 1.29e–34 < 5e–4 (see Supplementary Table 4).

**Table 1:** Per-patient statistics, stratified by gender and race/ethnicity (top: Site A, bottom: Site B).

Characteristic	Total count	% of full sample	Dropout count	% of dropout sample
Site A (*n* = 27,366)				
*Gender*				
Female	17246	63.02	5831	61.37
Male	10118	36.97	3671	38.63
*Race/Ethnicity*				
White	22564	82.45	7917	83.32
Black	1182	4.32	435	4.58
Asian	665	2.43	231	2.43
Hispanic	823	3.01	282	2.97
Other	2132	7.79	637	6.70
	Total mean	Total std	Dropout mean	Dropout std
Age (years)	46.9	15.7	48.31	15.56
Site B (*n* = 16,630)				
*Gender*				
Female	11771	70.78	4402	70.16
Male	4857	29.21	1871	29.82
*Race/Ethnicity*				
White	11980	72.04	4738	75.52
Black	1405	8.45	498	7.94
Asian	278	1.67	86	1.37
Hispanic	2003	12.04	566	9.02
Other	964	5.80	386	6.15
	Total mean	Total std	Dropout mean	Dropout std
Age (years)	49.0	14.9	50.19	14.56

Statistics associated with dropout are shown in the right columns. This table includes both primary care and psychiatrist providers.

### Model discrimination

**Fig. 2:**
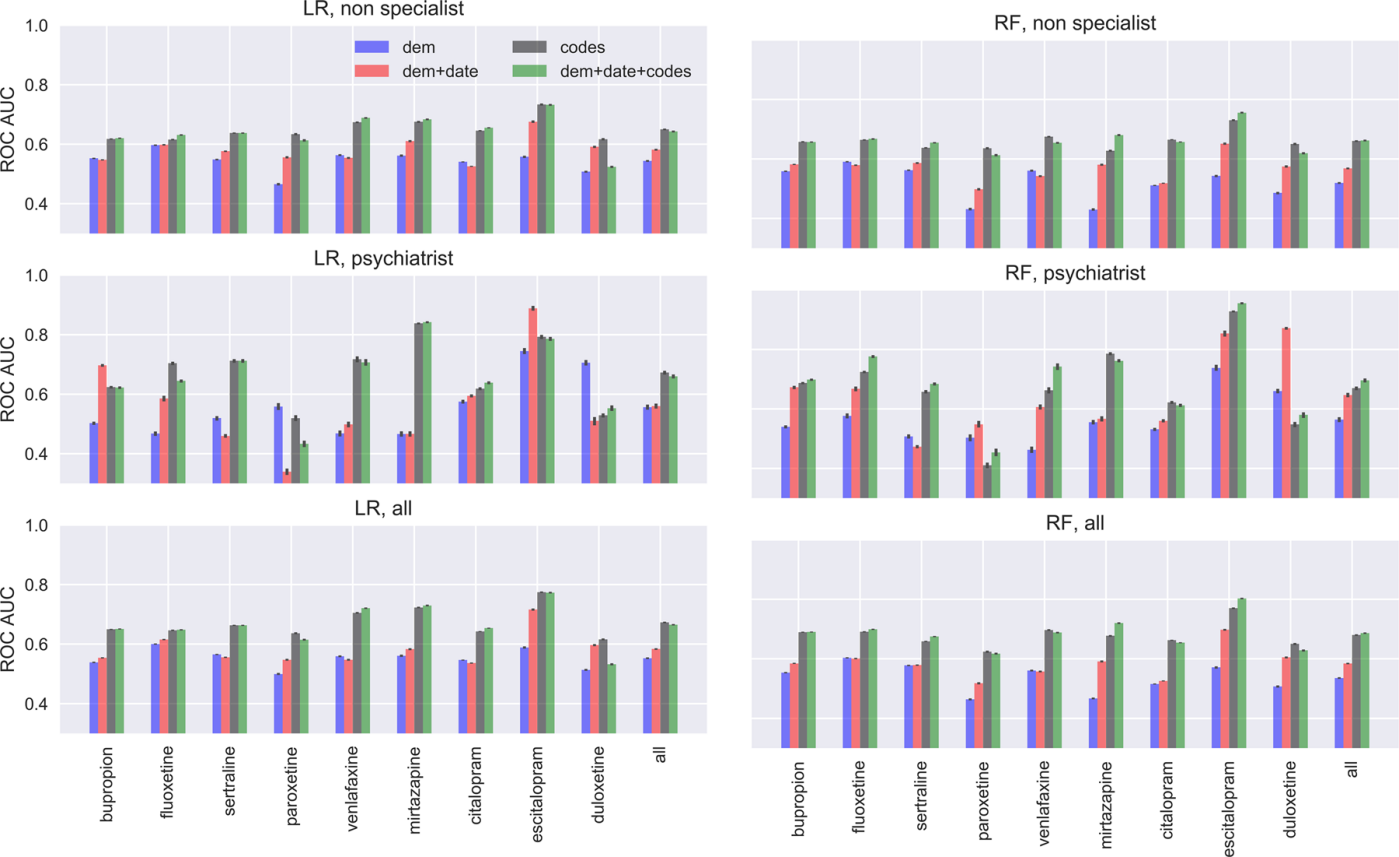
Test area under the curve (AUC) for the logistic regression (LR) classifier in the first column and random forest (RF) classifier in the second column, stratified by type of provider (from top to bottom: non-specialists, psychiatrists, all providers). Four different types of input data are considered: sociodemographic features (dem), date of prescription (date), and diagnostic/procedure codes (codes). Confidence intervals computed using 500 bootstraps.

Figure 2 shows the AUC for the LR and RF classifiers across individual medications and provider types. Both classifiers exhibit similar performances across medications; mean AUC was 0.67 [0.62–0.71] for LR and 0.69 [0.64–0.73] for RF, with best discrimination for escitalopram (AUC of 0.80), and poorest for paroxetine (AUC of 0.62). Compared with the baseline models (training with sociodemographic information alone, or sociodemographics plus the actual year of the prescription), incorporating EHR data increases discrimination substantially and significantly (median Welch’s *t*-test = −29.31; *p* = 5.34e–72 < 5e–4 for LR, and median Welch’s *t*-test = −26.08; *p* = 1.15e–65 < 5e–4 for RF) (Supplementary Table 3). AUC values are also greater in general for individuals treated by psychiatrists compared with non-psychiatrists: 0.66 vs 0.64 (median Welch’s *t*-test = −2.41; *p* = 3.79e–4 < 5e–4 for LR, and median Welch’s *t*-test = −6.38; *p* = 3.68e–15 < 5e–4 for RF). Detailed statistical validation can be found in Supplementary Table 3a, b. For categories of features most strongly associated with discontinuation in each model, see Supplementary Fig. 3.

### Prediction accuracy with respect to number of previous prescriptions

**Fig. 3:**
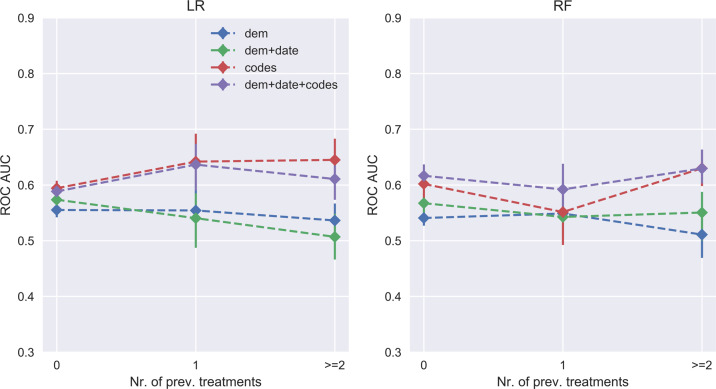
Test area under the curve (AUC) for the random forest (RF) classifier (same global classifier) stratified by number of previous distinct prescriptions. Stratification according to how many distinct treatments have already been tried in the past. AUC improves with the number of tried treatments in the past. Four different types of input data are considered: sociodemographic features (dem), date of prescription (date), and diagnostic/procedure codes (codes). Confidence intervals computed using 500 bootstraps.

We next examined the extent to which model performance depends on the number of prior treatment trials observed. Figure 3 presents this stratified analysis of AUC values according to prior treatments. In general, for models incorporating coded clinical data, AUC values are stable or improve with the number of previous documented treatments (see Supplementary Table 3c for statistical validation).

### Model replication at a second site

**Fig. 4:**
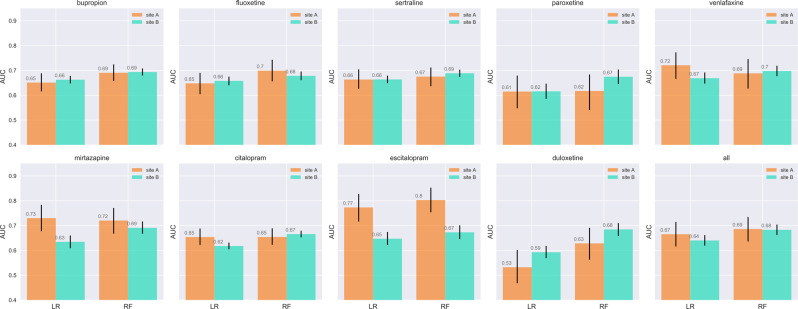
Test area under the curve (AUC) for the random forest (RF) and logistic regression (LR) classifiers given all patient history (sociodemographic information, date of prescription, diagnostic and procedure codes) for Site A and Site B, showing modest decrement in discrimination at Site B (mean 0.64 vs 0.67 for LR classifier, and 0.68 vs 0.69 for RF classifier). Confidence intervals computed using 500 bootstraps.

While Fig. 2 represents internal validation within Site A, Fig. 4 shows AUC values in the second health system, Site B, whose data were not used for training. AUCs using the best classifiers (RF) remain in the 0.67–0.70 range for all medications, with only modest change from Site A, providing support for the portability of these models. At this site, optimal model performance was achieved for venlafaxine with AUC of 0.70 [0.68–0.72], and poorest for paroxetine with AUC of 0.67 [0.65–0.70].

### Model calibration and lift

Supplementary Figure 4 shows a lift histogram of discontinuation rate for different deciles of prescriptions sorted according to the LR or RF classifier, indicating goodness of fit in both the original health system and the replication site. The top risk decile identified by both classifiers was associated with treatment discontinuation for nearly half of the observed encounters (e.g., 43.97% and 49.41% for LR and RF, respectively), compared with an average discontinuation rate of 24.92%—i.e., lift was >2. Similarly, Supplementary Fig. 5 shows calibration curves for the LR and RF classifiers, indicating promising calibration in both the original network (Site A) and the replication network (Site B).

## Discussion

In this investigation of 51,683 individuals with major depression across two academic medical center-based health systems, we identified predictive models that improve significantly on chance and on simple sociodemographic models in discriminating dropout risk by incorporating coded clinical features in LR and RF classifiers. Performance was only slightly diminished in a second health system, further supporting a lack of overfitting and the potential for portability. Models were also well-calibrated across both sites, with good correlation between predicted and observed discontinuation rates. We also observed that discrimination in these models was comparable among individuals with no prior treatment, one prior treatment, or two or more prior treatments; if anything, additional history tends to improve performance. Finally, we observed that discontinuation rates were greater among primary compared with specialty care settings, which likely reflects unmeasured differences in depression severity or other illness features, as multiple studies suggest that primary compared with specialty care for other diseases can yield similar outcomes (see, for example, Chai-Coetzer et al.^10^).

Our results are difficult to compare to prior work, including our own initial publication examining treatment-resistant depression in a large effectiveness study^11^. In particular, few studies have examined longitudinal health records and few have attempted to model dropout directly, despite the recognition that off-target effects of medications are useful in predicting clinical outcomes^12,13^. Other strategies aimed at personalized antidepressant treatment, including use of pharmacogenomic testing or other biomarkers, have yielded mixed results, among them a recent large negative study^14^. An important step in dissemination of any prediction model will be demonstration of improvement in outcomes using randomized, double-blind trials.

A key question is how models of this kind and accuracy might be applied to improve the care of patients with major depressive disorder; the models described here could have at least two possible applications. In the first case, a clinician is ready to prescribe a given medication based upon standard guidelines or algorithms. In this case, the risk for discontinuation predicted by the machine-learned model might help in prioritizing interventions aimed at retention in treatment and adherence, including making follow-up phone calls, deploying mobile applications to improve adherence, or simply scheduling an earlier return visit. That is, a clinician or case manager might see the predicted risk of failure to return and select from a range of strategies to increase follow-up. Alternatively, these models might be applied in settings where there are multiple reasonable next-step treatment options, in the form of a decision support tool that ranks such options in terms of predicted follow-up probability. Here, all other things being equal, the clinician might prefer the medication with the lowest risk of treatment discontinuation for that patient. Our models require no additional data collection, so they are straightforward to apply in real time at the point of care.

Beyond targeting treatments, these models may be useful in the design of a new generation of EHR-linked clinical trials^15^. For example, trials may be stratified by discontinuation risk, or interventions aimed at high-risk individuals could be studied in that subgroup^16^. The interpretability of machine-learned models would be particularly important in this regard, as it facilitates identification of modifiable risk factors specific to a given intervention.

Several important limitations merit consideration. The first reflects reliance on coded clinical data: undoubtedly, additional patient-level variables not captured in these data would further improve prediction. Most notably, the role of concomitant psychotherapy cannot be characterized; although such psychotherapy is captured in coded data and can be incorporated in prediction, the type of therapy being delivered cannot be determined. Models using coded data represent a starting point, a baseline to be improved upon. In addition, while the present study includes an independent replication site, both hospitals are located in the same region with overlapping catchment areas. Thus, it will be important to pursue further studies in other regions, including non-US health systems. An advantage of the code-only model is that it should be readily translatable to most US and European health systems.

We also note that while predictions are made for individual medications, we cannot exclude the possibility that some of the prediction is not treatment-specific per se. That is, some of the features may simply reflect overall dropout risk rather than risk with a given medication. More generally, these analyses do not seek to capture reasons for discontinuation, which are likely to be multi-factorial. They do not examine which adverse effects might contribute to dropout, or other relevant factors including cost, availability of transportation, and symptomatic worsening. We simply consider the face-valid question: which patients are most likely to receive a single antidepressant prescription, and then not return for follow-up? Undoubtedly, consideration of additional patient-level features could also improve prediction. As such, this work should be considered to complement, rather than replace, patient self-report and passive measures as outcome prediction tools.

With these limitations in mind, these results nonetheless provide a first step toward personalization of antidepressant treatment on the basis not of efficacy, but of tolerability. A key strength of the present study, beyond demonstrating portability, is a common model applicable to both primary and specialty care settings. Along with efforts to integrate other predictors, this work should encourage others to pursue modeling of this simple but important outcome. True personalization may be less likely to arrive with discovery of a single critical biomarker, but rather with incremental efforts to improve upon chance in the ability to stratify outcomes.

## Supplementary information

Supplementary Information

Supplementary Figure 1

Supplementary Figure 2

Supplementary Figure 3

Supplementary Figure 4

Supplementary Figure 5

Supplementary Table 1

Supplementary Table 2

Supplementary Table 3

Supplementary Table 4
