# Supplementary material for: Predicting treatment dropout after antidepressant initiation
Source: Transl Psychiatry. 2020 Feb 6;10:60. doi: 10.1038/s41398-020-0716-y (PMC7026064; doi:10.1038/s41398-020-0716-y)
Supplement: Supplementary file 1 — Supplementary Information [file 41398_2020_716_MOESM1_ESM.docx]

**Title:** Predicting treatment dropout after antidepressant initiation

**Authors:** Melanie F. Pradier, PhD; Thomas H. McCoy Jr, MD; Michael Hughes, PhD; Roy H. Perlis, MD MSc; Finale Doshi-Velez, PhD

**Supplementary Information**

Supplementary Table 1. List of 9 most-prescribed primary antidepressant medications. Antidepressant categories include SSRI: selective serotonin reuptake inhibitor, SNRI: serotonin and norepinephrine reuptake inhibitor, Other ADs: other antidepressants. File format: .xlsx

Supplementary Table 2. List of Current Procedural Terminology (CPT) codes used to determine whether the patient was having psychiatry-related encounters. If the patient was having psychiatry-related encounters after the last antidepressant prescription, we did not classify that prescription as causing disengagement with psychiatric treatment, even if the prescription length was short. File format: .xlsx

Supplementary Table 3. Statistical Validation of Area Under the Curve (AUC) values. (a) t-tests for AUC values for models with only demographic information and prescription date versus model given all patient history, including codes from the electronic health record (EHR); (b) t-test for AUC values for models given all patient history for psychiatrist providers versus non-specialists; (c) t-test for AUC values for first prescription versus subsequent prescriptions. File format: .xlsx

Supplementary Table 4. Statistical Validation of dropout rates using the Chi-square test. (a) Per-site statistical significance of dropout rates across different medications (all providers). Differences on dropout rates across medications are statistically significant. (b) Per-medication statistical significance between psychiatrists and non-specialist providers (both sites). Dropout rates among psychiatrist providers are statistically significantly lower than dropout rates among primary care providers. File format: .xlsx

Supplementary Figure 1. Diagram of cohort definition and number of subjects excluded by criteria in defining the study cohort. Exclusion by “other meds” refers to patients that received prescriptions for less common medications that are outside of this study. File format: .pdf

Supplementary Figure 2. Example of patient history showing evidence of dropout after day 225 from index antidepressant prescription. File format: .png

Supplementary Figure 3. Classification of top-10 features per medication selected by the logistic regression classifier according to International Classification of Diseases, Ninth Revision (ICD9)/Current Procedural Terminology (CPT) hierarchy of codes (level 1). Blue color corresponds to ICD9 codes, magenta color corresponds to CPT codes, and green color corresponds to medications. From top to bottom, a) logistic regression (positively correlated), b) logistic regression (negatively correlated), c) random forest. File format: .png

Supplementary Figure 4. Average lift histogram for the logistic regression classifier (first column) and random forest classifier (second column) across the 9 studied antidepressants in Site A (first row) and Site B (second row). Prescriptions are sorted according to their predicted probability of discontinuation. Confidence intervals computed using 500 bootstraps. File format: .png

Supplementary Figure 5. Average calibration curves for the logistic regression classifier (first column) and random forest classifier (second column) across the 9 studied antidepressants in Site A (first row) and Site B (second row). File format: .png
