# Supplementary material for: Predicting treatment dropout after antidepressant initiation
Source: Transl Psychiatry. 2020 Feb 6;10:60. doi: 10.1038/s41398-020-0716-y (PMC7026064; doi:10.1038/s41398-020-0716-y)
Supplement: Supplementary file 2 — Supplementary Figure 1 [file 41398_2020_716_MOESM2_ESM.pdf]

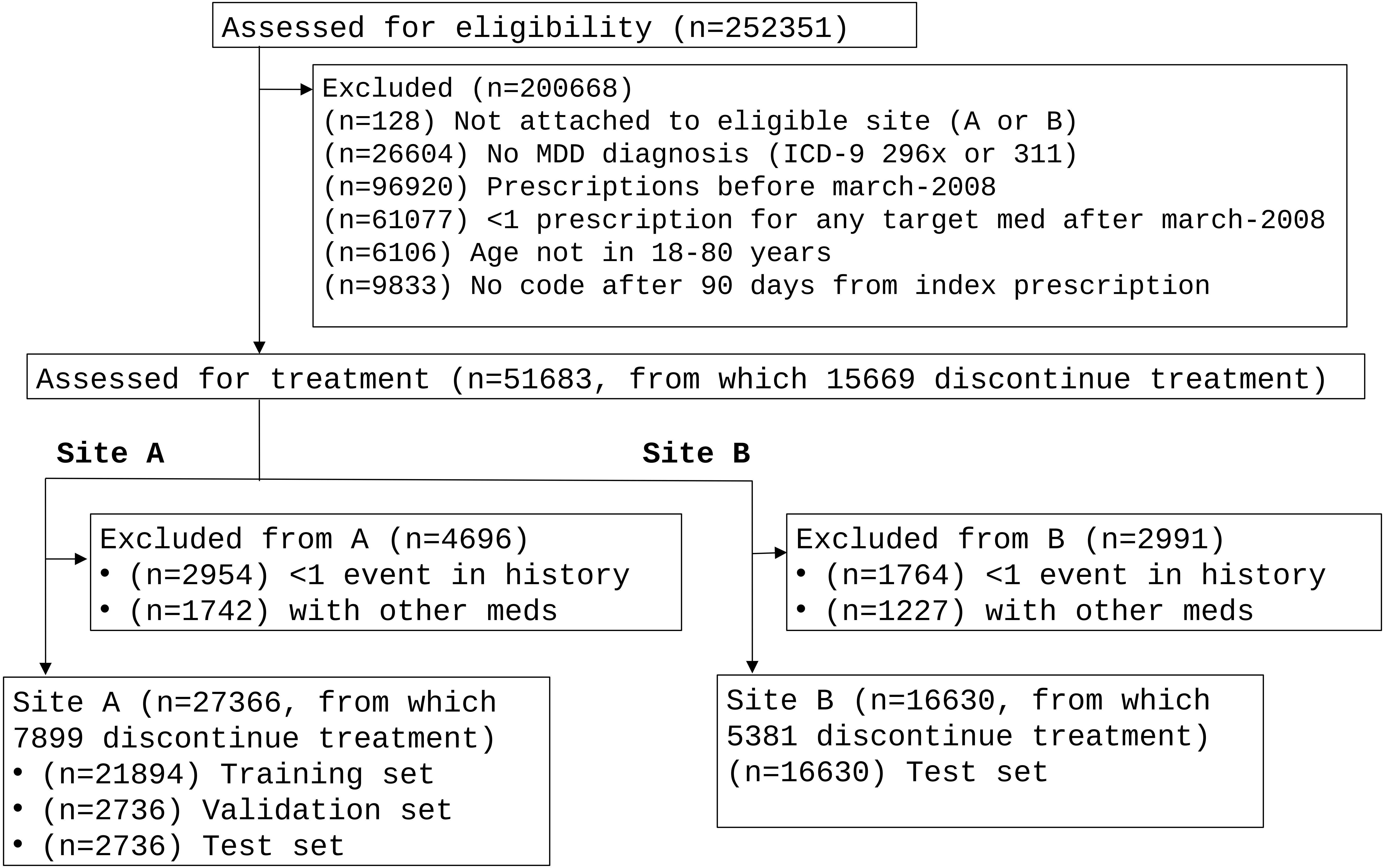
Assessed for eligibility (n=252351)
Excluded (n=200668)
(n=128) Not attached to eligible site (A or B)
(n=26604) No MDD diagnosis (ICD-9 296x or 311)
(n=96920) Prescriptions before march-2008
(n=61077) <1 prescription for any target med after march-2008
(n=6106) Age not in 18-80 years
(n=9833) No code after 90 days from index prescription
Assessed for treatment (n=51683, from which 15669 discontinue treatment)
Site A
Site B
Excluded from A (n=4696)
• (n=2954) <1 event in history
• (n=1742) with other meds
Excluded from B (n=2991)
• (n=1764) <1 event in history
• (n=1227) with other meds
Site A (n=27366, from which 7899 discontinue treatment)
• (n=21894) Training set
• (n=2736) Validation set
• (n=2736) Test set
Site B (n=16630, from which 5381 discontinue treatment)
(n=16630) Test set
