# Supplementary figures and images for: Predicting treatment dropout after antidepressant initiation
Source: Transl Psychiatry. 2020 Feb 6;10:60. doi: 10.1038/s41398-020-0716-y (PMC7026064; doi:10.1038/s41398-020-0716-y)

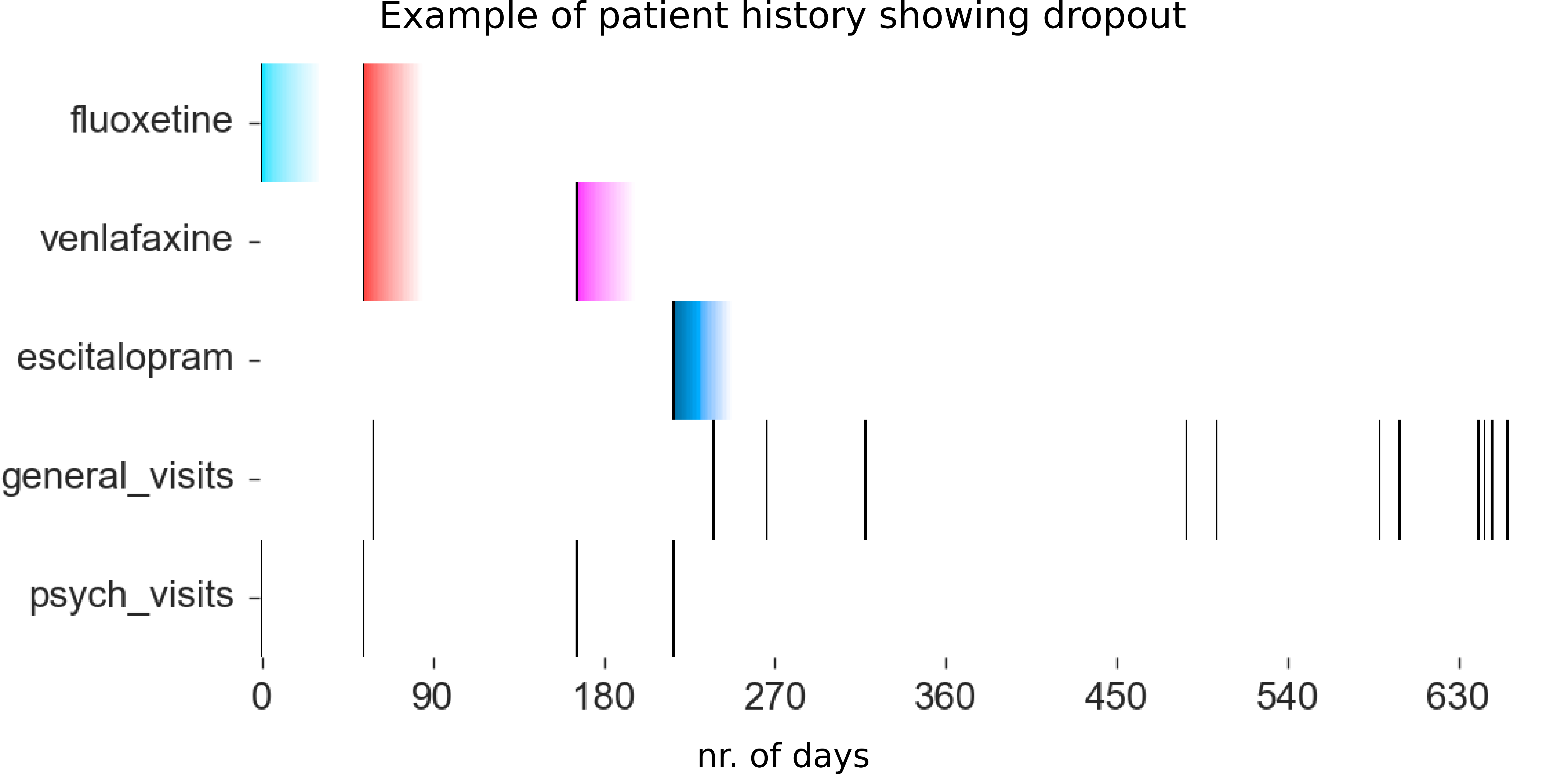

Supplement: Supplementary file 3 — Supplementary Figure 2 [file 41398_2020_716_MOESM3_ESM.png]

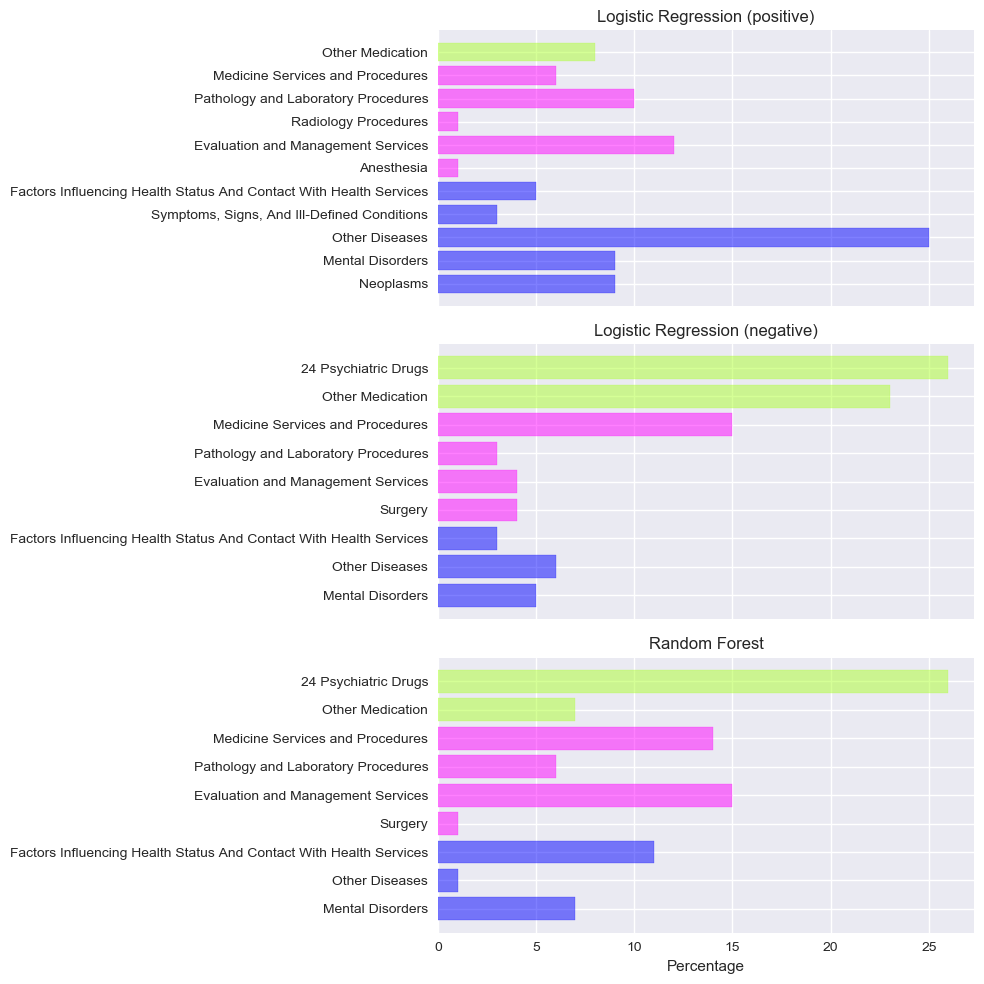

Supplement: Supplementary file 4 — Supplementary Figure 3 [file 41398_2020_716_MOESM4_ESM.png]

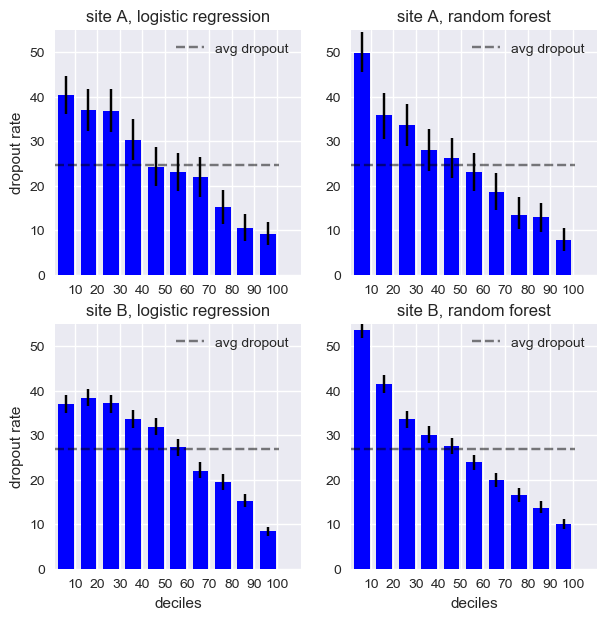

Supplement: Supplementary file 5 — Supplementary Figure 4 [file 41398_2020_716_MOESM5_ESM.png]

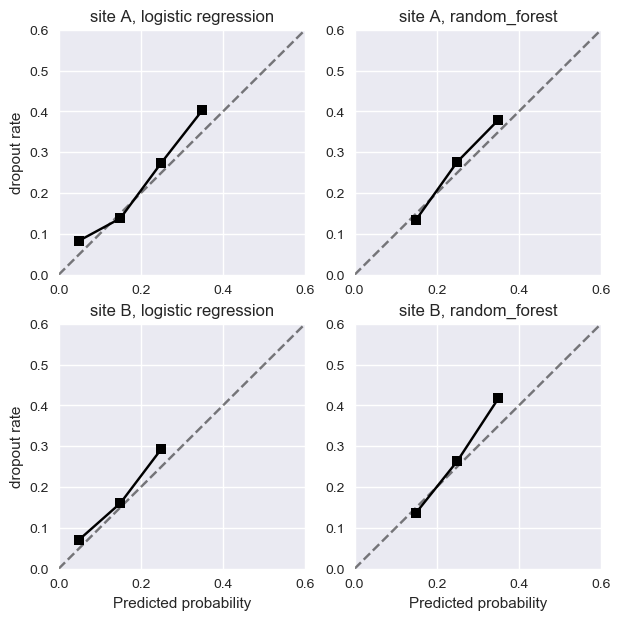

Supplement: Supplementary file 6 — Supplementary Figure 5 [file 41398_2020_716_MOESM6_ESM.png]
